# Association between motor symptoms of Parkinson’s disease and swallowing disorders

**DOI:** 10.1007/s10072-023-07238-1

**Published:** 2023-12-06

**Authors:** Masahiro Nakamori, Megumi Toko, Hidetada Yamada, Yuki Hayashi, Kai Ushio, Kohei Yoshikawa, Azusa Haruta, Aya Hiraoka, Mineka Yoshikawa, Toshikazu Nagasaki, Yukio Mikami, Hirofumi Maruyama

**Affiliations:** 1 Department of Clinical Neuroscience and Therapeutics, Hiroshima University Graduate School of Biomedical and Health Sciences, Hiroshima, Japan; 2 Department of Rehabilitation Medicine, Hiroshima University Hospital, Hiroshima, Japan; 3 Department of Advanced Prosthodontics, Hiroshima University Graduate School of Biomedical and Health Sciences, Hiroshima, Japan; 4 Department of Oral and Maxillofacial Radiology, Hiroshima University Graduate School of Biomedical and Health Sciences, Hiroshima, Japan

**Keywords:** Parkinson’s disease, Dysphagia, Motor symptoms, UPDRS, Videofluoroscopic examination

## Abstract

**Background:**

Parkinson’s disease (PD) presents with motor symptoms that hinder physical activity. This study aimed to thoroughly investigate swallowing dysfunction in patients with PD using videofluoroscopy (VF) and the Movement Disorder Society (MDS)-Unified PD Rating Scale (UPDRS) sub-scores.

**Methods:**

This study was part of an intervention project to evaluate the effectiveness of cervical percutaneous interferential current stimulation in patients with Hoehn and Yahr stages 2–4 PD. Baseline data, including swallowing-related indicators such as VF, were obtained and compared to the MDS-UPDRS sub-scores including rigidity, tremor, postural instability/gait difficulty, and limb scores.

**Results:**

Twenty-seven patients were included in this study. In the VF analysis, laryngeal penetration/aspiration, oral cavity residue, epiglottic vallecular residue, and pharyngeal residue were observed with remarkable frequency. The multivariate analysis revealed that the mean rigidity score of UPDRS was an independent and significantly correlated factor with laryngeal penetration/aspiration during the ingestion of 10 mL of water (odds ratio 1.294, 95% confidence interval 1.035–1.617; *p* = 0.024).

**Conclusion:**

This study revealed a correlation between muscle rigidity and laryngeal penetration or aspiration risk. The detailed comparative analysis of various individual PD symptoms and swallowing disorders was substantial, which enabled early detection of the risk of swallowing disorder and the implementation of appropriate measures.

**Trial registration number:**

jRCTs062220013.

## Introduction

Neurological disorders frequently result in dysphagia, increasing susceptibility to aspiration pneumonia and significantly affecting patient prognosis and outcomes. This leads to increased medical and caregiving costs. Parkinson’s disease (PD) is one of the most prevalent neurological disorders, and its incidence is rapidly increasing globally [1]. Aspiration pneumonia is the principal cause of mortality in patients with PD. Consequently, evaluating and formulating suitable and effective interventions for preventing pneumonia is paramount. PD causes several swallowing disorders, including anomalies in the oral cavity, pharyngeal transport [2], delayed swallowing reflex [3], and residual pharyngeal material [4].

Primary symptoms of PD include bradykinesia, muscular rigidity, and tremors [5]. Various clinical manifestations have been reported, and PD is now understood to be a systemic disorder encompassing non-motor symptoms as well. A heterogeneous array of diverse manifestations is observed even when considering only motor symptoms. Consequently, clinical symptoms, disease progression, and associated issues differ significantly among patients with PD. The complexity of swallowing difficulties is believed to arise from this diversity; thus it is difficult to evaluate this phenomenon comprehensively. Nevertheless, research investigating the relationship between the motor symptoms of PD and swallowing difficulties is exceedingly limited.

We are currently conducting a project investigating the effect of cervical percutaneous electrical interferential current stimulation on swallowing in PD [6]. Interferential current stimulation activates peripheral nerves in the throat without discomfort [7]. This approach potentially alleviates dysphagia and has shown positive effects on saliva production [8], swallowing frequency, and airway sensitivity [7]. As a conceptual foundation of this exploratory research, we investigated the correlation between the subtypes of motor symptoms in PD and swallowing dysfunction using videofluoroscopy (VF), the gold standard evaluation method.

This study aimed to thoroughly investigate swallowing dysfunction in PD by comparing VF swallowing results to the Movement Disorder Society (MDS)-Unified PD Rating Scale (UPDRS) subscores, which assess the severity of various PD symptoms [9].

## Materials and methods

### Ethics approval, registrations, and patient consent

This research was approved by the Certified Review Board of Hiroshima University (Hiroshima, Japan) (approval number: CRB6180006) and conducted in accordance with the national government’s regulations following the Helsinki Declaration of 1964. The study was registered with jRCT (jRCTs062220013). Written informed consent was obtained from all participating patients.

### Study design and protocol

This study comprised cross-sectional exploratory research that is part of the project in which we investigated the effect of cervical percutaneous electrical interferential current stimulation on swallowing. The project protocol has been published previously [6]. This study was conducted at the Hiroshima University Hospital. We enrolled patients who were clinically diagnosed with probable or confirmed PD according to the Movement Disorder Society criteria [5] and possessed the ability to provide informed consent. Additionally, they were required to be at Hoehn and Yahr stages 2–4 at the time of enrollment and provide written informed consent. The inclusion criteria were as follows: receiving consistent levodopa dose for > 1 month and age > 19 years < 86 years. Individuals meeting any of the following criteria were not considered for the study: having a pacemaker or implantable defibrillator; undergoing deep brain stimulation; currently pregnant or attempting to conceive; and a diagnosis or history of head or neck cancer, active pneumonia, or past swallowing rehabilitation. Participants’ motor symptoms and swallowing dynamics were examined using baseline data collected before an intervention.

### Videofluoroscopy

An X-ray imaging device (Ultimax-i; Canon Medical System Corporation, Tochigi, Japan) was used to conduct the tests while the patients were seated. The trials involved 3 and 10 mL of water mixed with 30%^w^/_w_ barium contrast medium (Barytester A240 Powder ®, Fushimi Pharmaceutical Co. Ltd., Kagawa, Japan). The patients were instructed to ingest this mixture, delivered via a syringe directly to the floor of the mouth. Employing an X-ray system, we captured images from various angles: forward, towards the lips, backward to the pharyngeal wall, upward to the nasal cavity, and downwards to the upper esophageal sphincter. The recordings were obtained at a rate of 30 frames/s and stored on a DVD. To prevent fatigue phenomena, these investigations outlined here were conducted at the beginning of the videofluoroscopy, with breaks taken to prevent consecutive swallows.

Three experienced dentists (A Hiraoka, A Haruta, and MY), blinded to patient information, evaluated the videofluorographic recordings. Their expertise was in assessing the presence or absence of laryngeal penetration/aspiration and identifying the clearance or prevalence of residue in the oral cavity, vallecular area, and pharynx following a single swallow.

Additionally, they measured the time the bolus took to pass through specific anatomical landmarks and performed a temporal analysis. Two key parameters were calculated: laryngeal elevation delay time (LEDT) and pharyngeal delay time (PDT). LEDT refers to the duration between the bolus tip reaching the vallecula and the peak laryngeal elevation. An established LEDT threshold was set at 0.32 s [10]. In contrast, PDT, is the duration from when the bolus tip reaches the intersection of the lower border of the mandible and the base of the tongue until laryngeal elevation begins. In healthy adults, this interval ranges from 0 to 0.2 s [11].

Furthermore, we assessed whether there was a delay in the swallowing reflex. This was defined as the liquid remaining in the pyriform sinuses for more than 0.1 s (equivalent to 3 frames) before being swallowed [12]. The three observers engaged in thorough discussions and reached a consensus for each observation and measurement.

### Data acquisition

Two neurologists (MN and HY) performed the clinical evaluation and diagnosis. The recorded data included body mass index (BMI), grip strength, disease duration, alcohol consumption, smoking habits, MDS-UPDRS score [9], medication, Functional Oral Intake Scale (FOIS) score, Eating Assessment Tool-10 (EAT-10) score, and blood test values [13]. Previously reported methods were used to investigate cough reflex, tongue pressure, and peak expiratory flow [14–16]. The levodopa equivalent daily dose (LEDD) was calculated according to a recent study [17]. All evaluations were conducted in the On state. By referring to a previous study [18], data were extracted and calculated separately for the following components from the MDS-UPDRS Part 3 to analyze motor symptom subtypes: the mean value of the rigidity (3.3 a–e), tremor (3.15–3.18), postural instability and gait difficulty (PIGD) (3.9–3.13), and limb (3.4–3.8) scores.

### Statistical analyses

Data are expressed as mean ± standard deviation or median (minimum, maximum) for continuous variables and frequencies and percentages for discrete variables. Univariate analyses were conducted using methods such as single regression analysis, contingency tables, analysis of variance, and logistic regression analysis to investigate the data related to the findings of VF for swallowing 3 and 10 mL of water, as well as temporal analyses. In this process, we included the mean values of rigidity scores, tremor scores, PIGD scores, and limb scores, and as mentioned, UPDRS Part 3, in the analyses. Subsequently, we explored the associations between various VF findings and the corresponding UPDRS subscores. Initially, we conducted univariate analyses and extracted factors with *p*-values < 0.10. These factors were then subjected to a multivariate analysis. Statistical analyses were performed using JMP statistical software version 16 (SAS Institute Inc., Cary, NC, USA). Appropriate statistical tests, such as the χ2 test, Mann–Whitney U test, or unpaired *t*-test, were employed to assess intergroup variances. Statistical significance was set at *p* < 0.05.

## Results

In this study, 27 participants were enrolled, and baseline data were obtained. The participant demographics are presented in Table 1. Additionally, detailed data, including temporal analysis of the VF conducted by swallowing 3 and 10 mL of water, are listed in Table 2. Laryngeal penetration/aspiration, oral cavity, epiglottic vallecular, and pharyngeal residues were found at a remarkable frequency. Although there was a slightly higher frequency of abnormal findings during swallowing 10 mL of water than during swallowing 3 mL of water, no statistically significant difference was observed. However, LEDT and PDT were almost within the normal range when swallowing 3 mL of water, in which the number of patients who exhibited deviations from the standard values of LEDT and PDT were five and two, respectively. Although an unpaired t-test indicated no statistically significant difference for swallowing 10 mL compared to 3 mL of water, a trend of extended LEDT was observed. The number of patients who had deviations from the standard values of laryngeal LEDT and PDT were 14 and 2, respectively. Using the χ2 test, the number of patients with prolonged LEDT when swallowing 10 mL of water was significantly higher than that when swallowing 3 mL of water (*p* = 0.010). However, LEDT was not associated with the UPDRS subscores, including the neck rigidity subscore. For swallowing 3 mL of water, five patients (18.5%) had swallowing reflex delays, and for swallowing 10 mL of water, four (14.8%) had swallowing reflex delays. However, this was not frequent.

**Table 1:** Patient characteristics

	*n* = 27
Age, years	72.4 ± 5.9
Sex (females), *n* (%)	10 (37.0)
Duration, years	6 (1, 20)
Body mass index, kg/m^2^	21.5 ± 2.9
Alcohol consumption, *n* (%)	3 (11.1)
Current smoking, *n* (%)	3 (11.1)
Hoehn and Yahr stage	3 (2, 4)
UPDRS score (total)	41 (19, 76)
UPDRS score (part 3)	24 (10, 50)
Mean value of rigidity scores × 10 (3.3 a-e)	14 (0, 24)
Mean value of tremor scores × 10 (3.15–3.18)	0 (0, 14)
Mean value of PIGD scores × 10 (3.9–3.13)	12 (2, 32)
Mean value of limb scores × 10 (3.4–3.8)	7 (2, 22)
Levo-dopa, mg	354 ± 197
LEDD, mg	568 ± 384
Maximum handgrip strength, kg	24.6 ± 6.1
FOIS	7 (6, 7)
EAT-10 < 3, *n* (%)	13 (48.1)
Tongue pressure, kPa	30.5 ± 8.4
Peak expiratory flow, L/min	198.6 ± 82.3
Cough test 5 times/min, *n* (%)	4 (14.8)

*UPDRS* Unified Parkinson’s Disease Rating Scale, *PIGD* postural instability and gait difficulty, *LEDD* Levodopa equivalent daily dose, *FOIS* Functional Oral Intake Scale, *EAT-10* Eating Assessment Tool-10. Data are expressed as mean ± standard deviation or median (minimum, maximum) for continuous variables, and frequencies and percentages for discrete variables

**Table 2:** Videofluoroscopy findings after 3 and 10 mL of water intake

	3 mL	10 mL
Laryngeal penetration or aspiration, *n* (%)	11 (40.7)	14 (51.9)
Oral cavity residue, *n* (%)	22 (81.5)	25 (92.6)
Epiglottic vallecula residue, *n* (%)	18 (66.7)	22 (81.5)
Pharyngeal residue, *n* (%)	15 (55.6)	17 (63.0)
Swallowing reflex delay, *n* (%)	5 (18.5)	4 (14.8)
Laryngeal elevation delay time, s	0.220 ± 0.144	0.387 ± 0.693
Pharyngeal delay time, s	0.017 ± 0.158	-0.070 ± 0.254

Data are expressed as mean ± standard deviation, and frequencies and percentages for discrete variables

An investigation into the correlation between VF findings during the ingestion of 3 and 10 mL of water and the patient's background revealed that for swallowing 10 mL of water, a connection was observed between the mean value of rigidity scores and laryngeal penetration/aspiration. Following the univariate analysis, the factors associated with laryngeal penetration/aspiration (*p* < 0.10) included BMI, UPDRS Part 3, and the mean value of the rigidity scores. To address the confounding effects of the UPDRS Part 3 and the mean value of rigidity scores, separate multivariate analyses involving BMI were conducted. Consequently, the mean value of rigidity scores was identified as an independent and significantly correlated factor with laryngeal penetration/aspiration during the ingestion of 10 mL of water (odds ratio 1.294, 95% confidence interval 1.035–1.617, *p* = 0.024) (Table 3). No significant association was observed between LEDT and UPDRS scores or their subscores, including the neck rigidity subscore.

**Table 3:** Factors associated with laryngeal penetration or aspiration for 10 mL water intake using videofluoroscopy

	Univariate analysis			Multivariate analysis			
	Laryngeal penetration or aspiration			Model 1		Model 2	
	( +) *n* = 14	(-) *n* = 13	*p*-value	odds	95% CI	odds	95% CI
Age, years	74.1 ± 6.2	70.6 ± 5.2	0.121				
Sex (females), n (%)	4 (28.6)	6 (46.2)	0.345				
Duration, years	7.5 (1, 13)	6 (2, 20)	0.922				
Body mass index, kg/m2	20.4 ± 2.5	22.6 ± 2.9	0.053	0.615	0.382–0.988	0.727	0.511–1.034
Alcohol consumption, n (%)	1 (7.1)	2 (15.4)	0.496				
Current smoking, n (%)	1 (7.1)	2 (15.4)	0.496				
Hoehn and Yahr stage	3 (2, 3)	3 (2, 4)	0.883				
UPDRS score (total)	42 (19, 76)	36 (21, 76)	0.382				
UPDRS score (part 3)	29 (14, 48)	18 (10, 50)	0.069			1.067	0.981–1.160
Mean value of rigidity scores × 10 (3.3 a–e)	16 (10, 24)	12 (0, 20)	0.043*	1.294	1.035–1.617		
Mean value of tremor scores × 10 (3.15–3.18)	1 (0, 14)	0 (0, 7)	0.152				
Mean value of PIGD scores × 10 (3.9–3.13)	13 (4, 28)	10 (2, 32)	0.172				
Mean value of limb scores × 10 (3.4–3.8)	8.5 (2, 17)	6 (2, 22)	0.661				
Levo-dopa, mg	361 ± 204	346 ± 196	0.852				
LEDD, mg	562 ± 340	575 ± 440	0.934				
Maximum handgrip strength, kg	24.6 ± 6.8	24.6 ± 5.5	0.986				
FOIS	7 (6, 7)	7 (7, 7)	0.374				
EAT-10 < 3, n (%)	5 (35.7)	8 (61.5)	0.180				
Tongue pressure, kPa	30.7 ± 9.0	30.3 ± 7.9	0.918				
Peak expiratory flow, L/min	190.4 ± 87.0	207.4 ± 79.5	0.601				
Cough test 5 times/min, n (%)	3 (21.4)	1 (7.7)	0.315				

Data are expressed as mean ± standard deviation or median (minimum, maximum) for continuous variables, and frequencies and percentages for discrete variables

Model 1: multivariate analysis of body mass index and mean value of rigidity scores (3.3 a–e). Model 2: multivariate analysis of body mass index and UPDRS part 3 score

*mean *p* < 0.05

*CI* confidence interval, *UPDRS* Unified Parkinson’s Disease Rating Scale, *PIGD* postural instability and gait difficulty, *LEDD* Levodopa equivalent daily dose, *FOIS* Functional Oral Intake Scale, *EAT-10* Eating Assessment Tool-10

## Discussion

Patients with PD exhibit a diverse range of neurological symptoms, including motor manifestations. Swallowing difficulties among patients with PD also vary. This study examined the correlation between swallowing and each subscore for the main motor symptoms in patients with PD. The results indicated that the frequency of laryngeal penetration or aspiration was increased with increasing muscular rigidity severity.

This study demonstrated that muscle rigidity was associated with a higher risk of aspiration than other motor symptoms; however, this tendency was not observed during swallowing 3 mL of water but rather during swallowing 10 mL of water. No significant results were obtained regarding the effect of swallowing smaller amounts of water. Furthermore, during the swallowing of 10 mL of water, an increasing number of patients with PD exhibited delayed laryngeal elevation to the peak. LEDT refers to the duration between the bolus tip reaching the vallecula and peak laryngeal elevation [10]. This phenomenon was also pronounced when the amount of water swallowed was greater. Although a significant correlation with UPDRS subscores was not observed, it is plausible that prolonging laryngeal elevation time during the swallowing of larger volumes, owing to the bradykinesia and muscle rigidity characteristic of PD was a consistent outcome. Therefore, patients with PD and pronounced muscle rigidity should be particularly cautious during swallowing and avoid a larger bolus amount.

PD symptoms are categorized into limb and axial symptoms, with axial symptoms strongly associated with falls and swallowing difficulties [19]. In this study, we also examined the PIGD subscore extracted from UPDRS Part 3; however, no significant correlation was found. Previous studies have reported a relationship between falls and swallowing difficulties [19]; although, discrepancies in the results could be attributed to differences in scoring methods. Nevertheless, in this study, the advantage was extracting scores from the UPDRS, the gold standard for PD assessment, and conducting swallowing evaluations using VF.

Conversely, no significant correlations were found between UPDRS scores, including subscores, and measures such as tongue pressure and peak expiratory flow. Some systematic reviews and meta-analyses suggest relations among age, tongue pressure, and hand grip [20, 21]. In conditions such as stroke, amyotrophic lateral sclerosis, and sarcopenia, strong correlations have been observed between disease severity scores and tongue pressure, which are also significantly associated with aspiration and pneumonia development [14, 22, 23]. However, these conditions are also associated with paralysis. PD generally does not lead to paralysis but presents with smooth movement impairment primarily characterized by bradykinesia, making strength evaluation methods insufficient for assessing aspiration risk. Exploring and developing simple screening methods and combining these approaches are considered crucial tasks in addressing swallowing evaluation and PD management.

This study had several limitations. First, the study was conducted at a single site, warranting subsequent investigations across multiple facilities and a larger sample size. Second, a notable challenge arose from the limited patient pool available for scrutiny in this intervention-oriented project. The primary focus was on cervical interferential current stimulation to enhance the cough reflex assessment, which dictated the sample size based on prior research and existing literature. Nonetheless, to conduct a comprehensive scrutiny and analysis of swallowing disorders in patients with diverse PD symptoms, a considerable patient cohort must be included. Therefore, further patient recruitment is required to facilitate future research.

## Conclusion

This study demonstrated a correlation between muscle rigidity and aspiration risk. The detailed comparative analysis of various individual symptoms of PD and swallowing disorders was substantial. The findings of this study can contribute to a thorough understanding of swallowing disorders in patients with PD and the possibility of precise interventions; thus, further research is warranted.

## Data Availability

The data supporting the findings of this study are available from the corresponding author upon request.
